# Haematological response in experimental human *Plasmodium falciparum* and *Plasmodium vivax* malaria

**DOI:** 10.1186/s12936-021-04003-7

**Published:** 2021-12-20

**Authors:** Stephen D. Woolley, Louise Marquart, John Woodford, Stephan Chalon, Joerg J. Moehrle, James S. McCarthy, Bridget E. Barber

**Affiliations:** 1 QIMR Berghofer Medical Research Institute, 300 Herston Road, Brisbane, QLD 4006 Australia; 2 Centre of Defence Pathology, Royal Centre for Defence Medicine, Joint Hospital Group, ICT Building, Birmingham Research Park, Vincent Drive, Birmingham, UK; 3 Present Address: Clinical Sciences Department, Liverpool School of Tropical Medicine, Pembroke Place, Liverpool, UK; 4 Medicines for Malaria Venture, 20 Route de Pre-Bois, PO Box 1826, 1215 Geneva, Switzerland; 5 Present Address: Laboratory of Malaria Immunology and Vaccinology, NIAID, National Institutes of Health, Bethesda, USA; 6 Present Address: The Peter Doherty Institute for Infection and Immunity, The University of Melbourne and the Royal Melbourne Hospital, Melbourne, VIC Australia

**Keywords:** *Plasmodium falciparum*, Induced blood-stage malaria, CHMI, VIS, Malaria, Anaemia

## Abstract

**Background:**

Malaria-associated anaemia, arising from symptomatic, asymptomatic and submicroscopic infections, is a significant cause of morbidity worldwide. Induced blood stage malaria volunteer infection studies (IBSM-VIS) provide a unique opportunity to evaluate the haematological response to early *Plasmodium falciparum* and *Plasmodium vivax* infection.

**Methods:**

This study was an analysis of the haemoglobin, red cell counts, and parasitaemia data from 315 participants enrolled in IBSM-VIS between 2012 and 2019, including 269 participants inoculated with the 3D7 strain of *P. falciparum* (Pf3D7), 15 with an artemisinin-resistant *P. falciparum* strain (PfK13) and 46 with *P. vivax*. Factors associated with the fractional fall in haemoglobin (Hb-FF) were evaluated, and the malaria-attributable erythrocyte loss after accounting for phlebotomy-related losses was estimated. The relative contribution of parasitized erythrocytes to the malaria-attributable erythrocyte loss was also estimated.

**Results:**

The median peak parasitaemia prior to treatment was 10,277 parasites/ml (IQR 3566–27,815), 71,427 parasites/ml [IQR 33,236–180,213], and 34,840 parasites/ml (IQR 13,302–77,064) in participants inoculated with Pf3D7, PfK13, and *P. vivax,* respectively. The median Hb-FF was 10.3% (IQR 7.8–13.3), 14.8% (IQR 11.8–15.9) and 11.7% (IQR 8.9–14.5) in those inoculated with Pf3D7, PfK13 and *P. vivax*, respectively, with the haemoglobin nadir occurring a median 12 (IQR 5–21), 15 (IQR 7–22), and 8 (IQR 7–15) days following inoculation. In participants inoculated with *P. falciparum*, recrudescence was associated with a greater Hb-FF, while in those with *P. vivax*, the Hb-FF was associated with a higher pre-treatment parasitaemia and later day of anti-malarial treatment. After accounting for phlebotomy-related blood losses, the estimated Hb-FF was 4.1% (IQR 3.1–5.3), 7.2% (IQR 5.8–7.8), and 4.9% (IQR 3.7–6.1) in participants inoculated with Pf3D7, PfK13, and *P. vivax*, respectively. Parasitized erythrocytes were estimated to account for 0.015% (IQR 0.006–0.06), 0.128% (IQR 0.068–0.616) and 0.022% (IQR 0.008–0.082) of the malaria-attributable erythrocyte loss in participants inoculated with Pf3D7, PfK13, and *P. vivax*, respectively.

**Conclusion:**

Early experimental *P. falciparum* and *P. vivax* infection resulted in a small but significant fall in haemoglobin despite parasitaemia only just at the level of microscopic detection. Loss of parasitized erythrocytes accounted for < 0.2% of the total malaria-attributable haemoglobin loss.

**Supplementary Information:**

The online version contains supplementary material available at 10.1186/s12936-021-04003-7.

## Background

Malaria remains a major cause of mortality and morbidity worldwide, with 229 million cases and 409,000 reported deaths in 2019 [1]. Furthermore, it is likely that the disruption of services due to the current COVID-19 pandemic may significantly increase the number of malaria cases and malaria deaths [2]. Malaria-associated anaemia is one of the most important complications of malaria [3–9]. Hospital-based studies have demonstrated that severe anaemia can occur in patients infected with *Plasmodium falciparum*, *Plasmodium vivax* and *Plasmodium malariae*, and in all three species increases the risk of death [3]. More recently, cross-sectional community surveys have demonstrated that asymptomatic and sub-microscopic infections are also associated with a high risk of anaemia, contributing significantly to the overall burden of malaria-associated anaemia [10, 11].

Malaria-associated anaemia is multi-factorial. Although occurring in part due to the rupture of parasitized red blood cells (RBCs), the major contributor to malaria is the loss of unparasitized RBCs, with previous studies estimating that the ratio of the loss of parasitized to unparasitized RBCs is 1:8 in *P. falciparum* [12, 13] and 1:34 in *P. vivax* [14]*.* Potential contributors to this loss of unparasitized cells include increased free radical damage [15], the loss of erythrocyte surface complement regulatory proteins [16] and the production of anti-phosphatidylserine antibodies [17]. Dyserythropoiesis caused by bone marrow suppression secondary to the direct effects of the parasites as well as the effect of cytokines [18–21], and inflammation-induced iron deficiency [22], are other key contributors to malaria-associated anaemia. Shortened red cell lifespan following artesunate therapy [23] may also be contributory.

The induced blood-stage malaria (IBSM) volunteer infection model developed at QIMR Berghofer in 1995 has recently assumed a key role in anti-malarial drug development [24]. To date, over 400 participants have been enrolled in these studies at QIMR Berghofer, including 342 inoculated with the fully sensitive *P. falciparum* 3D7 strain, 15 with the K13 artemisinin resistant stain, and 46 with *P. vivax*. In these studies, parasitaemia is closely monitored with a highly sensitive quantitative PCR targeting the 18S rRNA gene, and frequent blood sampling occurs to monitor haematological parameters throughout the course of infection. Therefore, these studies provide a unique opportunity to investigate the haematological response that occurs in early *P. falciparum* and *P. vivax* blood-stage infection.

In this study, haematology data from 26 IBSM volunteer infection studies undertaken at QIMR Berghofer over the last ten years were analysed, in order to describe the haematological response to *P. falciparum* and *P. vivax* infection, and to evaluate factors associated with the fractional fall in haemoglobin. The haematological and parasitaemia data were also used to estimate the relative contribution of the loss of parasitized and unparasitized cells to the total malaria-attributable haemoglobin loss.

## Methods

### Study design and participants

Data were retrieved from the records of 315 participants enrolled in 26 IBSM studies conducted at QIMR Berghofer between 2012 and 2019 (Additional file 1: Table S1). Details of these studies have been published previously [25–47]. In brief, malaria-naïve participants were included if they were aged 18–55 years, had no significant co-morbidities or concurrent illness, and had haematology and biochemistry results at baseline that were within the protocol specified range. Participants were inoculated (day 0) with red blood cells parasitized with either *P. falciparum* 3D7 (Pf3D7; n = 254), an artemisinin-resistant strain of *P. falciparum* with a defined mutation on the K13 propeller gene (PfK13; n = 15), or *P. vivax* (n = 46)*.* Parasitaemia was closely monitored at specified time-points throughout the studies by quantitative PCR (qPCR) targeting the species specific 18S ribosomal ribonucleic (rRNA) gene [48]. The studies involved evaluation of 18 different investigational antimalarial drugs, either alone or in combination (Additional file 1: Table S1). The day on which antimalarial treatment was administered ranged from day 7 to 8 for Pf3D7, day 9 for PfK13 and day 8 to 14 for *P. vivax*. For many of the studies, a sub-curative dose of the investigational drug was administered, resulting in recrudescence of parasitaemia to facilitate calculation of the drug minimum inhibitory concentrations. In all studies, participants were treated with a registered anti-malarial drug, typically artemether-lumefantrine, either at the time of recrudescence, or at the end of the study if recrudescence did not occur.

All studies were conducted in accordance with the Declaration of Helsinki and the International Committee of Harmonization Good Clinical Practice guidelines. Ethical approval for all studies was granted by the Human Research Ethics Committee at QIMR Berghofer. All participants provided informed written consent.

### Haematology measurements

Blood was taken for standard haematology laboratory testing during screening, prior to inoculation, prior to antimalarial treatment, and then at protocol defined time-points throughout the study. The maximum volume of blood taken within a 30-day period was 400 mL. For the purposes of analysis, baseline haemoglobin was defined as the last value prior to inoculation. Nadir haemoglobin was defined as the lowest haemoglobin from the day of first antimalarial treatment (termed ‘day of treatment’) onwards. The fractional fall in haemoglobin was the difference between baseline and nadir haemoglobin as a percentage of the baseline haemoglobin. The day of haemoglobin normalization was defined as the earliest day post-treatment at which the haemoglobin was equal to or greater than the baseline haemoglobin. The reticulocyte difference was defined as the difference between the baseline reticulocyte count prior to inoculation (day-1) and the final reticulocyte count at the end of study.

### Statistical analysis

Data was analysed using Stata V.16.0 and GraphPad Prism V.8.1. For categorical variables the number and frequency (%) were reported, and differences between groups compared using Pearson Chi-squared test or Fisher’s exact test. For normally distributed continuous variables the mean and standard deviation (SD) were reported and differences between groups were compared using the Student’s t-test or analysis of variance (ANOVA). Non-normally distributed continuous variables were summarized using the median and interquartile range (IQR) and differences between groups were compared using the Mann–Whitney and Kruskal–Wallis tests. The Wilcoxon signed-rank test was used to compare haemoglobin at baseline with haemoglobin at later time-points. In participants inoculated with *P. vivax*, the differences in haemoglobin parameters between those treated with artemether/lumefantrine and those treated with other drugs were compared using the non-parametric Dunn’s multiple comparison test. Correlations between parasitaemia and haematological parameters were evaluated using either Pearson’s or Spearman’s correlation, depending on distribution.

Parasitaemia parameters evaluated included the log_10_ transformed pre-treatment peak parasitaemia (PP_Pre_), peak parasitaemia (PP; the highest parasitaemia within 24 h of treatment), and the pre-treatment total parasite burden (TPB_Pre_). The TPB_Pre_ was determined using the area under curve (AUC) of the non-transformed 18S qPCR data from day 4 until the time of treatment. For *P. falciparum,* the TPB_Pre_ incorporated an adjustment to account for sequestered parasites, assumed to be approximately 25% of the total parasite burden at any given timepoint [49]. Linear regression analysis was used to evaluate the association between TPB_Pre_ or PP_Pre_ and the haematology parameters, adjusting for day of treatment and/or drug treatment. The parasite multiplication rates (PMR) and parasite reduction rates (PRR) were calculated as previously described [50, 51]. Parasite recrudescence was defined as a parasitaemia increase by > 1000 parasites/mL occurring more than 2 days post-treatment.

For each participant, the total erythrocyte loss was calculated by subtracting the red cell count (RCC) at the day of haemoglobin nadir from the baseline RCC. The malaria-attributable loss was then calculated by subtracting the estimated phlebotomy-related erythrocyte loss from the total erythrocyte loss. For this calculation, the individual total blood volume was calculated using Nadler’s method [52], with the phlebotomy-related erythrocyte loss estimated by multiplying the participant’s baseline RCC (× 10^12^/L) by the estimated total phlebotomy blood volume from inoculation until the median day of haemoglobin nadir (total 0.19 L/ total blood volume). To calculate the loss of parasitized RBCs as a proportion of the malaria-attributable erythrocyte loss, the TPB_Pre_ (parasites per mL) was divided by the malaria-attributable erythrocyte loss (see Additional file 1 for calculations). This calculation assumes that every parasitized erythrocyte is singly infected [53] and, therefore, the TPB_Pre_ is equal to the number of parasitized erythrocytes lost. The use of TPB_Pre_ also assumes that parasite replication does not continue after treatment.

## Results

### Participant’s demographics

The median ages of participants inoculated with Pf3D7 (n = 254), PfK13 (n = 15) and *P. vivax* (n = 46) were 24 (IQR 22–28), 23 (IQR 21–27) and 24 (IQR 21–31) years, respectively. The majority of participants in all three groups were male (73% in Pf3D7, 60% in PfK13 and 65% in *P. vivax*).

### Parasitaemia

The overall median PP_Pre_ for participants inoculated with *P. falciparum* was 10,277 (IQR 3566–27,815) parasites/mL (Table 1). This was higher in participants inoculated with PfK13 (71,427 [IQR 33,236–180,218] parasites/mL) compared to Pf3D7 (9,008 [IQR 3341–21,798] parasites/mL; p < 0.0001), possibly due to the PfK13 participants being treated later (day 9) than those inoculated with Pf3D7 (day 7 or 8). The median PP in the *P. falciparum* group was 20,218 (IQR 8350–55,570) parasites/mL, which was again higher in the PfK13 group (132,160 [IQR 69,160–309,057 parasites/mL]) compared to the 3D7 group (18,240 [IQR 7,901–49,995] parasites/ml; p < 0.0001). For participants inoculated with *P. vivax* the median PP_Pre_ was 34,840 (IQR 13,302–77,064) parasites/mL, and the PP 53,696 (IQR 15,934–102,635) parasites/mL, with both these values being significantly higher than those inoculated with *P. falciparum* (p < 0.001 for both comparisons). Recrudescence occurred in 96/269 (36%) participants in the *P. falciparum* group (15/15 [100%] in PfK13 and 84/254 [33%] in Pf 3D7), and 7/46 (15%) in the *P. vivax* group.

**Table 1:** Summary of haematology and parasite data

	Pf all (n = 269)	Pf 3D7 (n = 254)	Pf K13 (n = 15)	*P. vivax (*n = 46)	P-value (3D7 vs K13)	P-value (Pf all vs ***P. vivax*)**
Age, Years (Median, IQR)	24 (22–28)	24 (22–28)	23 (21–27)	24 (21–31)	0.38	0.87
Male Sex, n (%)	197 (73)	188 (74)	9 (60)	30 (65)	0.23	0.26
PP_Pre_, Parasites/mL (Median, IQR)	10,277 (3566–27,815)	9,008 (3341–21,798)	71,427 (33,327–180,218)	34,840 (13,302–77,064)	** < 0.0001**	** < 0.0001**
PP, Parasites/mL (Median, IQR)	20,218 (8350–55,570)	18,240 (7901–49,995)	132,160 (69,160–309,057)	53,696 (15,934–102,635)	** < 0.0001**	**0.0006**
TPB_Pre_, Parasites/mL (Median, IQR)	30,223 ^a^ (14,320–90,970)	28,366 ^a^ (12,637–75,571)	410,274 ^a^ (166,853–1,106,184)	31,345 (12,264–67,516)	** < 0.0001**	0.38
PRR (Median, IQR)	0.058 (0.045–0.081)	0.060 (0.045–0.082)	0.051 (0.045–0.055)	0.055 (0.048–0.068)	**0.040**	0.37
Baseline Hb, g/L (Median, IQR)	149 (139–156)	149 (141–156)	145 (136–153)	147 (139–158)	0.27	0.74
Day of Treatment Hb, g/L (Mean, SD)	139 (12.09)	140 (11.81)	136 (16.11)	140 (12.61)	0.22	0.57
Nadir Hb, g/L (Median, IQR)	132 (123–140)	132 (123–140)	127 (110–135)	129 (121–140)	**0.024**	0.22
Hb Fractional Fall, % (Median, IQR)	10.6 (7.9–13.8)	10.3 (7.8–13.3)	14.8 (11.8–15.9)	11.7 (8.9–14.5)	**0.001**	0.07
Day Post Treatment of Hb Nadir (Median, IQR)	12 (5–21)	12 (5–21)	15 (7–22)	8 (7–15)	0.66	0.11
Day Post Treatment of return to baseline Hb (Median, IQR)	28 ^b^ (22–37)	28 ^b^ (22–37)	^c^	20 (18–25)	^c^	** < 0.0001**
% Contribution of pre-treatment Hb drop to total drop in Hb (Median, IQR)	55 (23–78)	55 (24–78)	40 (11–76)	45 (27–56)	0.45	**0.020**
Baseline reticulocyte count, 10^9^/L (Median, IQR)	55^d^ (44–68)	54 ^d^ (43–68)	58 (49–76)	58 (46–74)	0.33	0.34
Maximum post-treatment reticulocyte count, 10^9^/L (Median, IQR)	68^d^ (54–90)	66 ^d^ (53–89)	84 (66–100)	72 ^e^ (58–89)	**0.048**	0.29
Reticulocyte difference, 10^9^/L (Median, IQR)	12 ^f^ (3–28)	11 ^g^ (1–27)	20 (10–33)	13 ^e^ (4–23)	**0.049**	0.99

n-number of participants; IQR- interquartile range; PP- peak parasitaemia; PP_Pre_- pre-treatment peak parasitaemia; TPB_Pre_- pre-treatment total parasite burden, determined using the AUC of the 18S qPCR data from day 4 until the time of treatment; PRR- parasite reduction ratio

^a^The TPB_Pre_ for the *P. falciparum* groups has been adjusted by a factor of 25% to account for sequestered parasites [50]; ^b^Pf all n = 267 and Pf3D7 n = 252 due to 2 participants not reaching haemoglobin baseline; ^c^none of the PfK13 participants’ haemoglobin returned to baseline; ^d^Pf all n = 254 and Pf3D7 n = 239; ^e^n = 44; ^f^n = 246; ^g^n = 231; Mann–Whitney test used unless otherwise stated

### Fall in haemoglobin following inoculation with* P. falciparum* and* P. vivax*

In all participants inoculated with *P. falciparum*, the median haemoglobin fell from a baseline of 149 (IQR 139–156) g/L to a nadir of 132 (IQR 123–140) g/L, representing a median fall of 17 g/L and a fractional fall of 10.6% (IQR 7.9–13.8%) (Table 1). The fall in haemoglobin prior to anti-malarial treatment accounted for a median 55% (IQR 23–78%) of the total fall. The haemoglobin nadir occurred a median of 12 (IQR 5–21) days post treatment and returned to baseline a median of 28 (IQR 22–37) days post treatment.

**Fig. 1:**
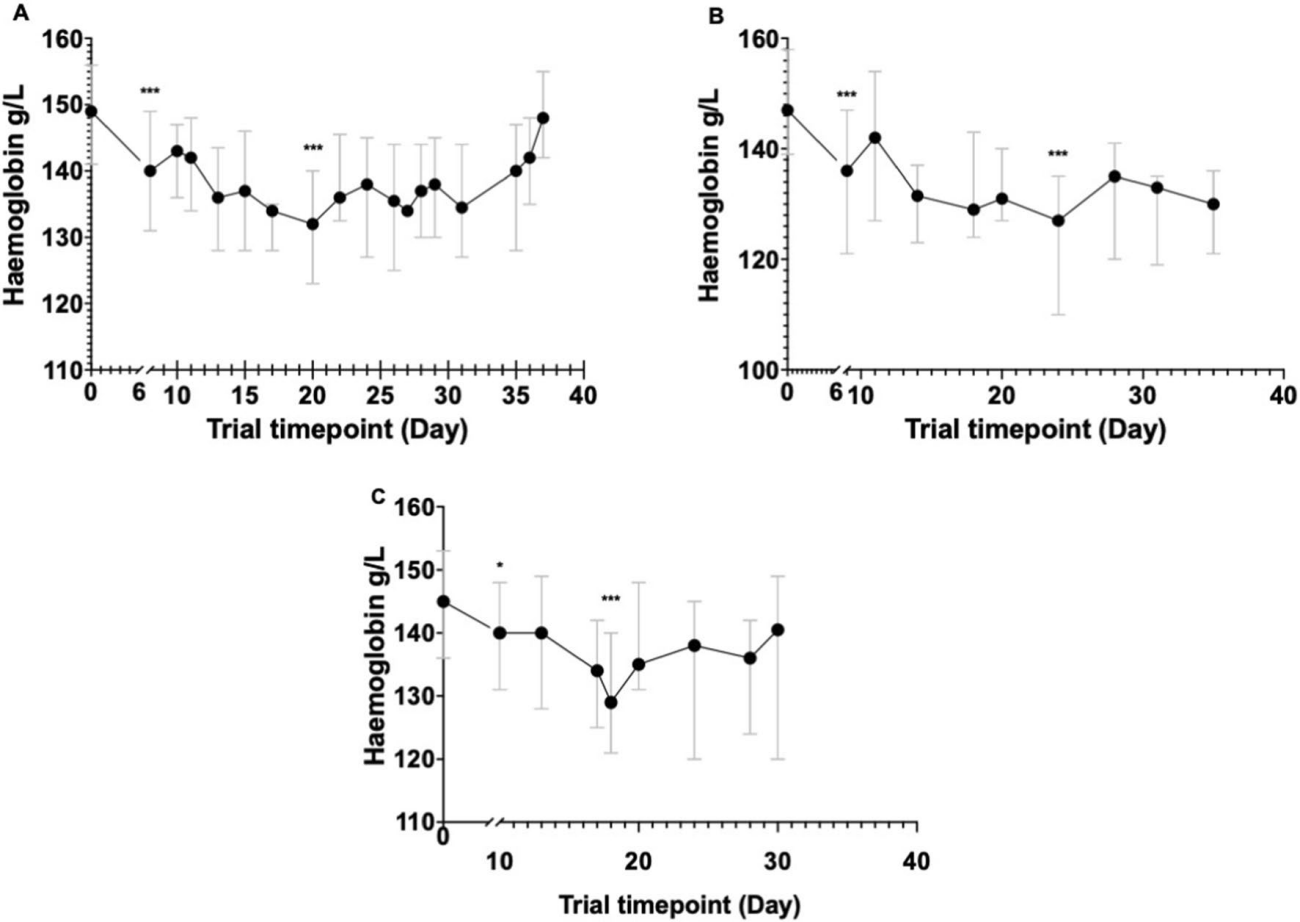
Changes in haemoglobin over time in participants inoculated with Pf3D7 (**A**), PfK13 (**B**) and *P. vivax* (**C**). Data points and error bars represent median and interquartile range, respectively. Values were compared to the values at baseline using the Wilcoxon matched-paired sign-rank test. *indicates a p value of 0.037, and *** indicates a p value of < 0.001, for comparisons with baseline haemoglobin

There was no significant difference between the median baseline haemoglobin between participants inoculated with Pf3D7 (149 g/L [IQR 141–156]) and those inoculated with PfK13 (145 g/L [IQR 136–153]; p = 0.27). In participants with PfK13, the haemoglobin fell by a median 18 (IQR 12–23) g/L compared to 17 (IQR 11–20) g/L in the Pf3D7 group, representing a median fractional fall of 14.8% (IQR 11.8—15.9%), compared to 10.3% (IQR 7.8–13.3%) in the 3D7 group (p = 0.001) (Fig. 1). The day of haemoglobin nadir occurred a median of 15 (IQR 7–22) days post treatment in those inoculated with PfK13 compared 12 (IQR 5–21) days in those inoculated with Pf3D7 (p = 0.66). In participants inoculated with the Pf3D7, the haemoglobin returned to baseline a median 28 (IQR 22–37) days post-treatment, while in the PfK13 group, the haemoglobin did not recover to baseline levels prior to the end of study in any of the 15 participants (Table 1).

In participants inoculated with *P. vivax*, the haemoglobin fell from a median of 147 (IQR 139—158) g/L to 129 (IQR 121–140) g/L, with a total median fall of 18 (IQR 14–21) g/L and a median fractional fall of 11.7% (IQR 8.9–14.5), which compared to 10.6% (IQR 7.9–13.8) in the *P. falciparum group* (p = 0.07). The median fall in haemoglobin prior to treatment contributed to 45% (IQR 27–56) of the total fall. The haemoglobin nadir occurred a median of 8 (IQR 7–15) days following treatment and returned to baseline levels a median of 20 (IQR 18–25) days following treatment.

### Factors influencing fall in haemoglobin

#### Recrudescence

In participants inoculated with *P. falciparum*, 99 (37%) participants who recrudesced had a fractional fall in haemoglobin of 12.0% (IQR 8.1—15.2; n = 99), compared to 10.2% (IQR 7.7–12.8; n = 170) in those who did not (p = 0.006). In the *P. vivax* group, the median haemoglobin fractional fall was similar in those who recrudesced (11.8% [IQR 8.7–16.4]; n = 7) compared to those who did not (11.7% [IQR 8.8–14.5; n = 39]; p = 0.95).

#### Age, sex and day of treatment

In the *P. falciparum* participants, there was no significant difference observed in the haemoglobin nadir or fractional fall in haemoglobin between age groups (Additional file 1: Table S4). In participants inoculated with *P. vivax*, a trend was observed towards a greater fractional fall of haemoglobin in the older age group (median 13.6% [IQR 10.8–14.6; n = 21] *vs* 10.5% [IQR 8.5–14.0; n = 25]; p = 0.09) (Additional file 1: Table S5), although this was not statistically significant.

Gender had no effect on the fractional fall in haemoglobin across all three species. Females had a lower median baseline haemoglobin than males (134 g/L [IQR 127–138; n = 88] vs 153 g/L [IQR 147–158; n = 227]; N = 315; p < 0.0001) as well as a lower haemoglobin nadir (117 g/L [IQR 111–123; n = 88] vs 136 g/L [IQR 130–142; n = 227]; N = 315; p < 0.0001).

In participants inoculated with Pf3D7, the day of treatment did not have any significant impact on the haematology parameters (Additional file 1: Table S6). However, for participants inoculated with *P. vivax*, where day of treatment was more variable, the median haemoglobin nadir was lower in participants treated on Day 10–14 (129 g/L [120–137]; n = 36), compared to those treated on Day 8 (140 g/L [133–144]; n = 10; p = 0.049). The median fractional fall in haemoglobin was also greater in those treated at Day 10–14 (12.4% [IQR 9.7–14.6]), compared to those treated on Day 8 (8.8% [IQR 7.2–11.7]; p = 0.030) (Additional file 1: Table S7). All participants in the PfK13 were treated on Day 9.

#### Drugs

**Fig. 2:**
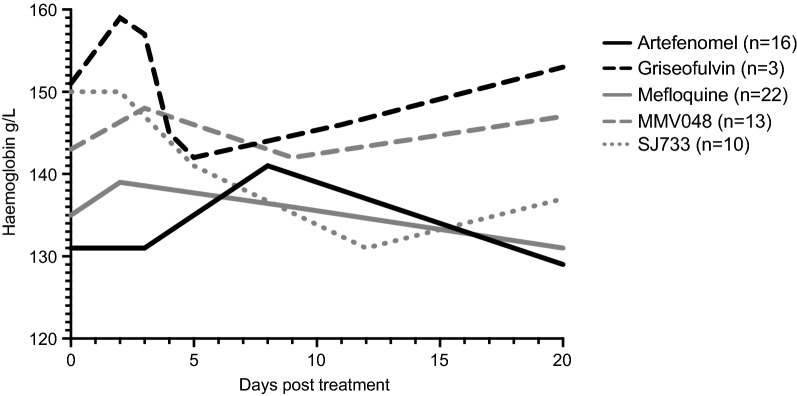
Median haemoglobin in participants inoculated with Pf3D7 who did not recrudesce, according to drug treatment

There were sixteen single or combination drugs assessed in the Pf3D7 participants (Table 2). However, substantial heterogeneity existed among these studies, precluding statistical analysis of drug effect on the fractional fall in haemoglobin. In particular, treatment was administered on day 7 in 10 studies, and on day 8 in 10 studies. The end of study (EOS) also varied, from Day 36 in the ZY-19489 study to Day 50 in the M5717 study. Recrudescence occurred more frequently in the ACT-451840 (7/8 participants) and SJ733 (6/16 participants) studies compared to the ferroquine study where no individuals recrudesced. Although statistical analysis was not performed, fractional falls in haemoglobin were numerically lowest in those treated with griseofulvin (8.5% [IQR 6.8–9.3; n = 3]), and highest in those treated with MMV048 (11.4% [IQR 8.4–14.2; n = 13) (Fig. 2).

**Table 2:** Haemoglobin parameters in participants inoculated with Pf 3D7 who did not recrudesce, according to drug treatment

Pf3D7 (n = 168) Drugs (n)	Nadir, g/L (median, IQR)	Fractional Fall (%, median, IQR)	Day post treatment of Hb Nadir (median, IQR)
ACT-451840 (1)	136	9.33	9
DSM265 (12)	135 (130–141)	10.9 (10.4–12.9)	21 (14–23)
DSM265 + Artefenomel (7)	115 (114–139)	10.6 (6.1–11.6)	21 (20–28)
Ferroquine (8)	129 (118–133)	11.3 (9.3–12.8)	20 (18–21)
Griseofulvin (3)	140 (136–146)	8.5 (6.8–9.3)	11 (4–20)
Cipargamin (4)	139 (133–144)	10.0 (8.0–13.0)	20 (12–23)
M5717 (20)	136 (132–142)	10.6 (8.5–12.9)	10 (6–14)
MMV048 (13)	138 (131–143)	11.4 (8.4–14.2)	9 (8–12)
Mefloquine (22)	127 (120–139)	9.1 (7.9–14.7)	4 (0–9)
Artefenomel (16)	125 (119–139)	8.6 (7.5–9.7)	8 (1–21)
Artefenomel + Piperaquine (21)	132 (127–140)	9.1 (7.0–13.2)	14 (0–27)
Piperaquine (23)	129 (120–140)	10.8 (6.3–13.0)	19 (0–23)
Piperaquine + Primaquine (2)	127–138	4.2–13.0	7–20
SJ733 (10)	136 (130–140)	9.8 (8.3–11.6)	12 (10–20)
ZY-19849 (6)	128 (122–137)	10.9 (5.5–11.6)	7 (3–8)

*n* number of participants, *IQR* interquartile range

Participants inoculated with *P. vivax* were treated with either chloroquine (n = 24), artefenomel (n = 8) or artemether/lumefantrine (n = 14), with no significant differences in the haemoglobin parameters observed between treatment groups (Additional file 1: Table S8). The PfK13 participants were all administered artesunate as the investigational medical product.

There was no correlation between speed of parasite killing (PRR) and fractional fall of haemoglobin in the overall *P. falciparum* and *P. vivax* groups (Additional file 1: Table S9). There was however a strong inverse correlation between PRR (r = − 0.57, p = 0.026) and the fractional fall in haemoglobin in the PfK13 group (Additional file 1: Table S10).

#### Effect of parasitaemia

In those inoculated with Pf3D7 and who did not experience recrudesce (n = 170), there were no correlations between any of the parasitaemia variables and haemoglobin nadir or fractional fall in haemoglobin, even after adjusting for drugs (Table 3). There were however strong correlations between PP_Pre_ (r = 0.66, p = 0.007) and PMR (r = 0.61, p = 0.016) and the fractional fall in haemoglobin in the PfK13 group, although all (15/15) PfK13 participants recrudesced (Additional file 1: Table S10).

**Table 3:** Correlations between parasite parameters and haemoglobin variables in *P. falciparum* and *P. vivax* in those who did not recrudesce

Correlation	Pf3D7 (n = 170)			*P. vivax* (n = 39)		
	r-value	Unadjusted P-value ^a^	Adjusted P-value ^b^	r-value	Unadjusted P-value ^a^	Adjusted P-value ^c^
PP_Pre_ and Haemoglobin nadir	0.06	0.44	0.82	− **0.47**	**0.002**	**0.001**
PP_Pre_ and Fractional Fall of Haemoglobin	− 0.05	0.49	0.43	**0.32**	**0.045**	**0.041**
TPB_Pre_ and Haemoglobin nadir	0.07	0.39	0.99	− **0.57**	**0.0001**	**0.002**
TPB_Pre_ and Fractional Fall of Haemoglobin	− 0.01	0.89	0.78	**0.39**	**0.013**	**0.040**

*n* number of participants, *TPB*_*Pre*_ pre-treatment total parasite burden, *PP*_*Pre*_ Pre-treatment peak parasitaemia

^a^Pearson’s correlation (with PP_Pre_ and TBP log_10_ transformed); ^b^adjusted for drugs by including drug as an explanatory variable in a linear regression model; ^c^adjusted for drugs and by day of treatment by including drug and day of treatment as explanatory variables in a linear regression model

In participants inoculated with *P. vivax* and who did not experience recrudescence (n = 39), the PP_Pre_ correlated with the fractional fall in haemoglobin (r = 0.32, p = 0.045), with this correlation remaining significant when adjusted for drugs (p = 0.041). The TPB_Pre_ correlated with the haemoglobin nadir (r = − 0.57, p = 0.0001) and fractional fall in haemoglobin (r = 0.39, p = 0.013), with both correlations remaining significant after adjustment for drug (p = 0.0001 and p = 0.040, respectively) (Table 3).

#### Malaria-attributable erythrocyte loss and the contribution of parasitized erythrocytes to the malaria-attributable erythrocyte loss

In participants inoculated with *P. falciparum*, the median total erythrocyte loss from inoculation until the day of haemoglobin nadir was 0.5 (IQR 0.3–0.7) × 10^12^ erythrocytes/L, with this loss being greater in those inoculated with PfK13 (median 0.7 [IQR 0.6–0.8] × 10^12^/L) compared to Pf3D7 (median 0.5 [IQR 0.3–0.6] × 10^12^/L; p = 0.0005) (Table 4). The erythrocyte loss in those inoculated with *P. vivax* (median 0.5 [IQR 0.4–0.6] × 10^12^ erythrocytes/L) was similar to those inoculated with Pf3D7.

**Table 4:** Loss of infected erythrocytes as a percentage of total erythrocyte loss, in participants inoculated with Pf3D7, PfK13 and *P. vivax*

**Pf all (n = 267)**		**Median (IQR)**	**Pf 3D7 (n = 254)** **Median (IQR)**	**Pf K13 (n = 15)** **Median (IQR)**	***P. vivax* (n = 46)** **Median (IQR)**	**P-value** **(3D7 vs K13) **^**d**^	**P-value** **(Pf all vs *P. vivax*) **^**d**^
Baseline RCC (× 10^12^/L)		5.0 (4.7–5.8)	5.0 (4.7–5.8)	5.1 (4.6–5.2)	5.0 (4.7–5.2)	0.58	0.57
Nadir RCC (× 10^12^/L)		4.5 (4.2–4.8)	4.5 (4.2–4.8)	4.4 (3.8–4.6)	4.4 (4.0–4.8)	0.07	0.31
Total RCC Loss (× 10^12^/L)		0.5 (0.3–0.7)	0.5 (0.3–0.6)	0.7 (0.6–0.8)	0.5 (0.4–0.6)	**0.0005**	0.57
Malaria-attributable RCC loss (× 10^12^/L) ^a^		0.21 (0.04–0.36)	0.20 (0.04–0.33)	0.34 (0.12–0.50)	0.21 (0.04–0.33)	0.06	0.87
Malaria-attributable RCC loss/total RCC loss (%) ^b^		42 (13–51)	40 (13–55)	49 (20–63)	42 (13–55)	0.29	0.46
Loss of pRBC/total malaria-attributable RBC loss (%) ^c^		0.015 (0.006–0.060)	0.014 (0.005–0.039)	0.128 (0.068–0.616)	0.022 (0.008–0.082)	** < 0.0001**	0.19

*n* number of participants; *IQR* interquartile range

^a^Calculated by subtracting the estimated phlebotomy-related RBC loss from the total RBC loss. For this calculation, the phlebotomy-related RBC loss was estimated by multiplying the participant’s baseline RCC (× 10^12^/L) by the estimated total phlebotomy blood volume (0.19L) from day of inoculation to the median day of haemoglobin nadir; ^b^The percentage contribution of the malaria-attributable erythrocytes losses from the total erythrocyte losses was determined by dividing the malaria-attributable losses by the erythrocyte losses and multiplying by 100 to generate a percentage; ^c^The loss of pRBCs was equal to the TPB_Pre_, which was determined using the AUC of the 18S qPCR data from day 4 until the time of treatment. For *P. falciparum*, the TPB_Pre_ incorporated an adjustment to account for parasite sequestration, assumed to be an assumed approximately 25% at any given timepoint. This calculation assumed that each erythrocyte was singly infected, and that parasite replication did not continue following treatment; ^d^Mann–Whitney test of significance, not adjusted for multiple comparisons

The malaria-attributable erythrocyte loss was estimated to be a median 0.21 (IQR 0.04–0.36) × 10^12^/L in participants inoculated with *P. falciparum*, or ~ 42% of the total erythrocyte loss. Again, this loss was greater in the PfK13 group (median 0.34 [IQR 0.12–0.50] × 10^12^/L, or 49% of the total erythrocyte loss) compared to Pf3D7 group (median 0.20 [IQR 0.04–0.33] × 10^12^/L, or 40% of the total erythrocyte loss). In the *P. vivax* group, the median malaria-attributable erythrocyte loss was 0.21 (IQR 0.4–0.33) × 10^12^/L, similar to the erythrocyte loss in the Pf3D7 group (Table 4). Applying these percentages to the fractional fall in haemoglobin, the malaria-attributable fractional fall in haemoglobin was 4.1% (IQR 3.1–5.3) in those inoculated with Pf3D7 group, 7.2% (IQR 5.8–7.8) in those inoculated with PfK13, and 4.9% (IQR 3.7–6.1) in those inoculated with *P. vivax*.

In participants inoculated with *P. falciparum*, parasitized cells accounted for an estimated 0.015% (IQR 0.006–0.060%) of the malaria-attributable erythrocyte loss. This proportion was significantly higher in the PfK13 group (median 0.128 [IQR 0.068–0.616] %) compared to the Pf3D7 group (0.014 [IQR 0.005–0.039] %, p < 0.0001). In those inoculated with *P. vivax*, parasitized cells accounted for an estimated 0.022 (IQR 0.008–0.082) % of the malaria-attributable erythrocyte loss. The loss of parasitized cells as a percentage of the total malaria-attributable erythrocyte loss was associated with the TPB_Pre_ in participants inoculated with each species (*P. falciparum* r = 0.83, p < 0.001; *P. vivax* r = 0.63, p < 0.001) and with each *P. falciparum* inocula (Pf3D7 r = 0.81, p < 0.001; PfK13 r = 0.56, p = 0.035).

#### Reticulocyte response

**Fig. 3:**
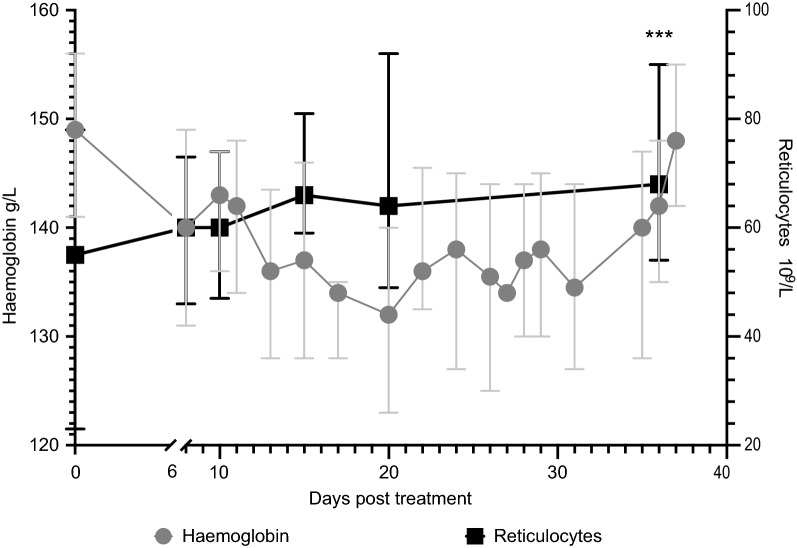
Median haemoglobin and reticulocyte count over time in participants inoculated with Pf3D7 (n = 254). Error bars represent interquartile range. Values were compared to the values at baseline using the Wilcoxon matched-paired sign-rank test. *** p < 0.0001 (for the difference between the reticulocyte count at end of study and the reticulocyte count at baseline)

In those inoculated with *P. falciparum*, the reticulocyte count increased from a median of 55 (IQR 44–68) × 10^9^/L at baseline to 68 (IQR 54–90) × 10^9^/L at EOS, representing a median 22% increase (p = 0.0001) (Table 1, Fig. 3). A similar increase was seen among those inoculated with *P. vivax*. There was a significant correlation between the reticulocyte response and total erythrocyte loss in the *P. falciparum* group (r = 0.13, p = 0.04; n = 240) but not in the in the *P. vivax* group (r = 0.08, p = 0.60; n = 44). In the *P. falciparum* group, there was a correlation between age and the reticulocyte response (r = 0.15, p = 0.019; n = 240), although this was not observed in the *P. vivax* group.

## Discussion

In this study, the haematological response to early experimental *P. falciparum* and *P. vivax* infection is described. The analysis demonstrates that in both *P. falciparum* and *P. vivax*, experimental infection results in an ~ 11% fractional fall in haemoglobin, approximately half of which occurs prior to treatment. The haemoglobin nadir occurred ~ 12 days after treatment in participants inoculated with *P. falciparum* and 8 days in participants inoculated with *P. vivax*, returning to normal by 28 days in *P. falciparum* and 20 days in *P. vivax*.

Volunteer infection studies are associated with frequent blood sampling, and it was estimated that phlebotomy-related losses accounted for ~ 60% of the total fractional fall in haemoglobin. Nonetheless, the malaria-attributable loss from early experimental malaria, with parasitaemias only just reaching the limit of detection by microscopy, still accounted for a fractional fall in haemoglobin of ~ 4% in Pf3D7, ~ 7% in PfK13 and ~ 5% in *P. vivax*, after accounting for phlebotomy related losses. It should be noted that in experimental malaria the duration of parasitaemia prior to treatment is only a few days, in contrast to endemic regions where low-level asymptomatic parasitaemia may persist in partially immune individuals for prolonged periods, thus the haemoglobin loss may be expected to be substantially greater. This is supported by recent studies from endemic regions, which have demonstrated a significant burden of anaemia associated with submicroscopic parasitaemia [10, 11].

In participants inoculated with *P. falciparum*, the fractional fall in haemoglobin was greater in those inoculated with an artemisinin-resistant PfK13 strain, compared to those inoculated with the artemisinin-sensitive Pf3D7 strain. Contributing factors that may have accounted for this greater fractional fall include a later day of treatment and higher parasitaemias in participants inoculated with PfK13 compared to Pf3D7, as well as the slower parasite reduction ratio, and the recrudescence that occurred in all of the PfK13 participants following treatment with artesunate. The day of haemoglobin nadir also occurred later in the PfK13 participants, and no participant recovered their haemoglobin prior to the end of study, again likely reflecting delayed parasite clearance and recrudescent parasitaemia. In the PfK13 participants the parasite reduction ratio was significantly and inversely correlated with the fall in haemoglobin, with slower parasite clearance associated with greater haemoglobin fall. Although this study was not designed to directly compare haemoglobin losses in participants inoculated with PfK13 vs Pf3D7, the results reported here are consistent with data from clinical studies reporting a greater incidence of anaemia in patients with drug-resistant *P. falciparum* [54, 55] and those given drugs with slower parasite clearance effects [56–59]. Thus, while this study did not allow direct comparison of the effect of different drugs on haemoglobin loss, the impact of parasite clearance on rates of anaemia should be considered in clinical trials evaluating antimalarials.

Consistent with the greater fall in haemoglobin in participants inoculated with PfK13, all of whom recrudesced, within the Pf3D7 group those who recrudesced also experienced a significantly greater fall in haemoglobin. These data are consistent with previous studies which have also demonstrated higher rates of anaemia associated with parasite recrudescence [13, 60].

It is also possible that the greater fractional fall in haemoglobin observed in participants inoculated with PfK13 may have been due in part to the fact that these participants were treated with artesunate. Post-artesunate anaemia is well described, and results from delayed clearance of once-infected erythrocytes [23]. However, it is unlikely that this would be a significant contributor to erythrocyte loss at such low parasitaemias, and no participant inoculated with PfK13 had elevated markers of haemolysis [44].

In participants inoculated with *P. vivax*, we found that delaying day of treatment was associated with a greater fall in haemoglobin. This is also consistent with clinical studies reporting correlations between the time since symptom onset and degree of anaemia [61–64], and highlights the importance of early initiation of treatment.

In participants inoculated with *P. vivax*, a correlation between parasitaemia (whether measured as peak parasitaemia, or pre-treatment total parasite burden) and the fractional fall in haemoglobin was observed. This is as expected, and consistent with clinical data demonstrating a correlation between parasitaemia and anaemia [63, 64]. Unexpectedly, this association was not observed in the much larger group of participants inoculated with Pf3D7. It is possible the heterogeneity of the Pf3D7 studies (with different treatment days and different drugs evaluated), together with the low parasitaemias, may have obscured any association between parasitaemia and fractional fall in haemoglobin.

In this study we attempted to quantify the loss of parasitized cells as a proportion of the overall malaria-attributable erythrocyte loss. Similar analyses have been conducted previously, including in clinical malaria in endemic regions [13], and in neurosyphilis patients receiving malariotherapy [12, 14]. In the former study, Price et al. [13] evaluated the haematological response to *P. falciparum* malaria in over 4000 children and adults in Thailand during 1990–1995, and estimated that parasitized cells accounted for 7.9% of the total malaria-attributable loss. A similar estimate was obtained by Jakeman et al. [12], who used data from neurosyphilis patients undergoing malariotherapy to estimate that *P. falciparum* parasitized cells accounted for ~ 10.5% of erythrocytes lost. In neurosyphilis patients inoculated with *P. vivax*, Collins et al. [14] estimated that only 2.9% of the reduction in haemoglobin was due to destruction of parasitized erythrocytes. In the current study involving very low parasitaemias, our analyses demonstrated that parasitized cells accounted for only 0.015% of erythrocytes lost in volunteers inoculated with Pf3D7, and 0.022% in those inoculated with *P. vivax*. This suggests that in submicroscopic infections, the relative contribution of the loss of unparasitized cells to malarial anaemia is likely much greater than that seen in higher parasitaemia infections.

The mechanisms mediating the loss of unparasitized cells at such low parasitaemias remain incompletely understood. In malaria volunteer infection studies, despite the low parasite counts, participants still experience a significant inflammatory response, with elevated levels of IFN-γ and IL-6 [65–67] potentially contributing to inhibition of erythropoiesis [68]. Additional contributors to the loss of unparasitized cells in acute clinical malaria include haemolysis, decreased red blood cell deformability, antibody and complement binding to erythrocytes, loss of complement regulatory proteins on the surface of unparasitized erythrocytes [69] and increase in splenic size [70] with associated splenic clearance of uninfected erythrocytes [71]. However, the role of these processes in low-parasitaemia infections such as volunteer infection studies has been more difficult to define [16].

This study had a number of limitations. First, there was substantial heterogeneity between and within the individual malaria volunteer infection studies, including the drug used, the day of treatment, and the study duration, making it difficult to account for factors associated with haemoglobin loss. Second, in the *P. vivax* infection studies, the calculation of total parasite burden prior to antimalarial treatment did not account for parasite sequestration. Recent studies have suggested that in chronic *P. vivax* infection, a very high proportion of parasitized erythrocytes are sequestered in the spleen [52], and it has also been shown that splenic accumulation may occur even in early *P. vivax* infection [70]. It is possible that this may in part explain the low contribution of peripheral parasitized cells to the malaria-attributable erythrocyte loss, in this study and in others [14]. Finally, this study was conducted in malaria-naïve healthy adults, and these data may not be applicable to children, or to adults in malaria-endemic regions where immunity maybe present.

In summary, this study demonstrates that a small but statistically significant fall in haemoglobin occurs in experimental malaria infection, despite parasitaemias that are only just at the level of microscopic detection. This study adds to studies from endemic regions reporting a significant burden of anaemia from asymptomatic and submicroscopic infections, highlighting the importance of treating these groups to reduce the overall burden of anaemia. Finally, this detailed description of the expected haemoglobin loss in malaria volunteer infection studies can be used as a baseline against which to compare the haemoglobin losses that may occur when new antimalarial drugs are evaluated in this model.

## Supplementary Information

**Additional file 1.** Additional tables.

## Data Availability

The datasets used and/or analysed during the current study are available from the corresponding author on reasonable request.

## Abbreviations

ANOVA: Analysis of variance
AUC: Area under the curve
EOS: End of study
HB-FF: Fractional fall in haemoglobin
HREC: Human research ethics committee
IBSM: Induced blood-stage malaria
IFN: Interferon
IQR: Interquartile range
MMV: Medicines Malaria Venture
Pf3D7: 3D7 strain of *P. falciparum*
PfK13: K13 artemisinin-resistant strain of *P. falciparum*
PMR: Parasite multiplication rate
PP: Peak parasitaemia
PP_Pre_: Pre-treatment peak parasitaemia
PRR: Parasite reduction ratio
pRBC: Parasitized red blood cell
qPCR: Quantitative Polymerase Chain Reaction
RBC: Red blood cell
RCC: Red cell count
RNA: Ribonucleic acid
SD: Standard deviation
TPB_Pre_: Pre-treatment total parasite burden
uRBC: Unparasitized red blood cell
VIS: Volunteer infection studies
