# Supplementary material for: Haematological response in experimental human Plasmodium falciparum and Plasmodium vivax malaria
Source: Malar J. 2021 Dec 20;20:470. doi: 10.1186/s12936-021-04003-7 (PMC8685492; doi:10.1186/s12936-021-04003-7)
Supplement: Supplementary file 1 — Additional file 1. Additional tables. [file 12936_2021_4003_MOESM1_ESM.docx]

**Additional Appendix**

**Additional file 1: Table S1. List of clinical trials**

| Clinical Trial ID | Drug/ Dose | Year | Inoculum | Day of Treatment | No of Subjects | Reference |
| --- | --- | --- | --- | --- | --- | --- |
| ACTRN12612000323820 | Mefloquine | 2012 | Pf 3D7 | 7 or 8 | 22 | [1] |
| ACTRN12612000814875 | Artefenomel | 2012 | Pf 3D7 | 7 or 8 | 24 | [2] |
| ACTRN12612001096842 | Artemether/lumefantrine | 2012 | *P. vivax* | 14 | 2 | [3] |
| ACTRN12613000533796 | DSM265 | 2013 | Pf 3D7 | 8 | 9 | [4] |
| ACTRN12613000669796 | Artemether/lumefantrine | 2013 | Pf 3D7 | 8 | 2 | [5] |
| ACTRN12613000565741 | Piperaquine | 2013 | Pf 3D7 | 7 or 8 | 24 | [6] |
| ACTRN12613000698774 | Griseofulvin | 2013 | Pf 3D7 | 8 | 5 | [7] |
| ACTRN12613001008718 | Artemether/lumefantrine | 2013 | *P. vivax* | 14 | 6 | [8] |
| ACTRN12613001040752 | Ferroquine | 2013 | Pf 3D7 | 8 | 8 | [9] |
| ACTRN12614000781640 | ACT-451840 | 2014 | Pf 3D7 | 7 | 8 | [10] |
| ACTRN12614000930684 | Artemether/lumefantrine | 2014 | *P. vivax* | 8 | 2 | [11] |
| NCT02281344 | MMV390048 | 2014 | Pf 3D7 | 7 | 6 | [12] |
| NCT02389348 | Artefenomel + DSM265 | 2015 | Pf 3D7 | 7 | 13 | [13] |
| NCT02431637 | Piperaquine | 2015 | Pf 3D7 | 7 | 6 | [14] |
| NCT02543086 | Cipargamin + Piperaquine | 2015 | Pf 3D7 | 7 | 8 | [15] |
| NCT02431650 | Piperaquine + Artefenomel or Primaquine | 2015 | Pf 3D7 | 7 | 11 | [14] |
| NCT02573857 | DSM265 (*P. falciparum*) and Artefenomel (*P. vivax*) | 2015 | Pf3D7/ *P. vivax* | 7 or 10 | 15 | [16,17] |
| ACTRN12616000174482 | Chloroquine | 2016 | *P. vivax* | 8 or 10 | 24 | [11] |
| NCT02867059 | SJ733 | 2016 | Pf 3D7 | 8 | 16 | [18] |
| NCT02783833 (Part B) | MMV390048 | 2016 | Pf 3D7 | 8 | 15 | [19] |
| ACTRN12617000244303 | Artesunate | 2017 | Pf K13 | 9 | 2 | [20] |
| NCT03261401 | M5717 | 2017 | Pf 3D7 | 8 | 22 | [21] |
| ACTRN12617001394336 | Artesunate | 2017 | Pf 3D7/Pf K13 | 8 or 9 | 22 | [20] |
| ACTRN12617001502325 | Artemether/lumefantrine | 2017 | *P. vivax* | 10 or 11 | 4 | [22] |
| NCT03542149 | Artfenomel + Piperaquine | 2018 | Pf 3D7 | 8 | 24 | [23] |
| ACTRN12619001215112 | ZY-19489 | 2019 | Pf 3D7 | 8 | 15 | [24] |

**Calculations**

1. Total RCC loss (x10^12^ cells/L) = *Baseline RCC – RCC at haemoglobin nadir*

The baseline RCC is the RCC on the day of inoculation. The haemoglobin nadir is the lowest haemoglobin from the day of first antimalarial treatment onwards.

1. Individual total blood volume in litres (using Nadler’s method)

Male= [0.3669 x (height in metres)^3^] + [0.03219 x (weight in kg)] + 0.6041

Female= [0.3561 x (height in metres)^3^] + [0.03308 x (weight in kg)] + 0.1833

1. Phlebotomy losses (x 10^12^ cells/L) = *Baseline RCC x (0.19/ individual total blood volume)*

The estimated total phlebotomy losses from inoculation to the median day of haemoglobin nadir was 0.19 L, which is divided by the individual total blood to give a proportion of the total blood volume. The volume is then multiplied against the baseline RCC to give the phlebotomy erythrocyte losses.

1. Malaria attributable losses (x 10^12^ cells/L) = *Total RCC Loss – Phlebotomy Loss*
2. Malaria attributable losses (cells/L) = *Malaria attributable losses x 10^12^*
3. Malaria attributable losses (cells/μL) = *Malaria attributable losses (cells/L)/10^6^*

Division by 10^6^ is the conversion factor from cells/L to cells/μL

1. TPB_PreSeq_/mL [for *P. falciparum* species only] = *TPB_Pre_ [calculated via AUC] x 1.33*

For *P. falciparum* species only, the TPB_Pre_ was adjusted for sequestered parasites, which is assumed to 25% of the TPB at any one time, therefore the TPB_Pre_ is multiplied by 1.33.

1. TPB_PreSeq_/μL or TPB_Pre_ for *P. vivax* = *TPB_PreSeq_ or TPB_Pre_ [P. vivax]/ 1000*

Conversion of the TPB_PreSeq_/μL or TPB_Pre_ from parasites/mL to parasites/μL, by dividing by 1000. This ensures both the malaria-attributable RBC losses and TPB_PreSeq_ or TPB_Pre_ are in cells/parasites per μL.

1. Loss of pRBC/total malaria-attributable RBC loss (%):

*TPB_PreSeq_ [P. falciparum] or TPB_Pre_ [P. vivax]* *x 100*

*Malaria attributable losses (cells/*μ*L)*

**Additional file 1: Table S2**. **Effect of recrudescence on haemoglobin parameters in all participants inoculated with *P. falciparum***

| Pf (n=269) | Recrudescence (n=99) | No recrudescence (n=170) | P-value ^a^ |
| --- | --- | --- | --- |
| Baseline Hb, g/L (Median, IQR) | 150  (141-157) | 148  (138-155) | 0.47 |
| Nadir Hb, g/L (Median, IQR) | 130  (123-141) | 133  (123-140) | 0.39 |
| Hb Fractional Fall, % (Median, IQR) | 12.0  (8.1-15.2) | 10.2  (7.7-12.8) | 0.006 |
| Day post treatment of Hb Nadir, Day (Median, IQR) | 14  (6-20) | 11  (4-21) | 0.74 |
| Day post treatment of return to baseline Hb, Day (Median, IQR) | 28  (22-36) | 29  (21-37) | 0.68 |

n, number of participants; SD, standard deviation; IQR, interquartile range.

a: calculated by Mann-Whitney test.

**Additional file 1: Table S3. Effect of recrudescence on haemoglobin parameters in participants inoculated with *P. vivax***

| *P. vivax* (n=46) | Recrudescence (n=7) | No recrudescence (n=39) | P-value ^a^ |
| --- | --- | --- | --- |
| Baseline Hb, g/L (Median, IQR) | 147  (136-152) | 136  (126-167) | 0.65 |
| Nadir Hb, g/L (Median, IQR) | 139  (125-141) | 129  (119-140) | 0.21 |
| Hb Fractional Fall, % (Median, IQR) | 11.8  (8.7-16.4) | 11.7  (8.8-14.5) | 0.95 |
| Day post treatment of Hb Nadir, Day (Median, IQR) | 15  (8-15) | 8  (7-16) | 0.38 |
| Day post treatment of return to baseline Hb, Day (Median, IQR) | 21  (18-33) | 20  (18-25) | 0.57 |

n, number of participants; SD, standard deviation; IQR, interquartile range.

a: calculated by Mann-Whitney test.

**Additional file 1: Table S4. Effect of age on the haemoglobin parameters in all participants inoculated with *P. falciparum***

| Pf 3D7 (n=254) | Ages (years) | | | |
| --- | --- | --- | --- | --- |
|  | **18-24 (n=143)** | **25-34 (n=102)** | **35+ (n=24)** | **P-value ^a^** |
| Nadir Hb, g/L (Median, IQR) | 133  (125-141) | 130  (120-139) | 134  (127-139) | 0.09 |
| Hb Fractional Fall, % (Median, IQR) | 10.2  (7.9-13.3) | 11.0  (8.2-14.4) | 10.4  (6.0-12.8) | 0.15 |
| Day post treatment of Hb Nadir, Day (Median, IQR) | 14  (5-21) | 10  (5-20) | 14  (7-20) | 0.16 |
| Day post treatment of Hb Return, Day (median, IQR) | 28  (22-36) | 28  (22-36) | 33  (27-43) | 0.30+ |

n, number of participants; IQR, interquartile range.

a: calculated by Kruskal-Wallis test

**Additional file 1: Table S5. Effect of age on the haemoglobin parameters in participants inoculated with *P. vivax***

| *P. vivax* (n=46) | Ages (years) | | |
| --- | --- | --- | --- |
|  | **18-24 (n=25)** | **25+ (n=21)** | **P-value ^a^** |
| Nadir Hb, g/L (Median, IQR) | 135  (125-141) | 124  (120-135) | 0.07 |
| Hb Fractional Fall, % (Median, IQR) | 10.5  (8.5-14.0) | 13.6  (10.8-14.6) | 0.09 |
| Day post treatment of Hb Nadir, Day (Median, IQR) | 8  (7-15) | 8  (5-16) | 0.56 |
| Day post treatment of Hb Return, Day (median, IQR) | 20  (18-21) | 19  (16-27) | 0.43 |

n, number of participants; IQR, interquartile range.

a: calculated by Mann-Whitney test

**Additional file 1: Table S6. Effect of Day of Treatment on the haemoglobin parameters in participants inoculated with Pf3D7**

| Pf 3D7 (n=254) | Day of Treatment | | |
| --- | --- | --- | --- |
|  | **7 (n=74)** | **8 (n=180)** | **P-value ^a^** |
| PP_Pre_, Parasites/mL (Median, IQR) | 7,329  (3,375-14,982) | 10,243  (3,275-28,546) | 0.05 |
| TPB_Pre_, Parasites/mL (Median, IQR) | 13,505  (4,261-26,364) | 44,877  (18,157-97,669) | <0.0001 |
| Nadir Hb, g/L (Median, IQR) | 130  (120-141) | 133  (125-140) | 0.49 |
| Hb Fractional Fall, % (Median, IQR) | 9.7  (7.2-12.8) | 10.6  (8.0-13.8) | 0.12 |
| Day post treatment of Hb Nadir, Day (Median, IQR) | 19  (6-21) | 10  (5-20) | 0.052 |
| Day post treatment of Hb Return, Day (median, IQR) | 28  (22-35) | 29  (22-38) | 0.15 |

n, number of participants; IQR, interquartile range; PP_Pre_, Peak parasitaemia pre-treatment; TPB_Pre_, Pre-treatment total parasite burden, adjusted by a factor of 25% for sequestered parasites.

a: calculated by Mann-Whitney test

**Additional file 1: Table S7. Effect of Day of Treatment on the haemoglobin parameters in participants inoculated with *P. vivax***

| *P. vivax* (n=46) | Day of Treatment | | |
| --- | --- | --- | --- |
|  | **8 (n=10)** | **10-14 (n=36)** | **P-value ^a^** |
| PP_Pre_, Parasites/mL (Median, IQR) | 6,480  (3,952-8,949) | 45,268  (30,437-82,905) | 0.003 |
| TPB_Pre_, Parasites/mL (Median, IQR) | 8,333  (4,690-10,895) | 45,557  (26,732-87,598) | <0.0001 |
| Hb Fractional Fall, % (Median, IQR) | 8.8  (7.2-11.7) | 12.4  (9.7-14.6) | **0.030** |
| Day post treatment of Hb Nadir, Day (Median, IQR) | 14  (7-20) | 8  (5-15) | 0.09 |
| Day post treatment of Hb Return, Day (median, IQR) | 20  (19-21) | 19  (18-27) | 0.34 |

n, number of participants; IQR, interquartile range; PP_Pre_, Peak parasitaemia pre-treatment; TPB_Pre_, Pre-treatment total parasite burden.

a: calculated by Mann-Whitney test

**Additional file 1: Table S8. Effect of drugs on the haemoglobin parameters in participants inoculated with *P. vivax***

| *P. vivax* (n=46) | Drug Classes | | | |
| --- | --- | --- | --- | --- |
|  | **Chloroquine**  **(n=24)** | **Artefenomel**  **(n=8)** | **Artemether/lumefantrine**  **(n=14)** | **P-value ^a^** |
| Nadir Hb, g/L (Median, IQR) | 129  (114-140) | 137  (128-140) | 129  (121-139) | 0.44 |
| Hb Fractional Fall, % (Median, IQR) | 11.3  (8.8-14.2) | 11.3  (9.6-14.4) | 13.8  (8.9-14.7) | 0.91 |
| Day post treatment of Hb Nadir, Day (Median, IQR) | 7  (6-18) | 11  (8-15) | 8  (3-14) | 0.56 |
| Day post treatment of Hb Return, Day (median, IQR) | 20  (18-21) | 19  (18-27) | 20  (14-28) | 0.96 |

n, number of participants; IQR, interquartile range.

a: calculated using Dunn’s test

**Additional file 1: Table S9. Spearman’s correlations between parasite parameters and haemoglobin variables in all participants inoculated with *P. falciparum* and *P. vivax* who did not recrudesce**

| Correlation | Pf All(n=170) | | *P. vivax* (n=39) | |
| --- | --- | --- | --- | --- |
|  | **r-value** | **P-value** | **r-value** | **P-value** |
| PP_Pre_ and Haemoglobin nadir | 0.07 | 0.37 | -0.51 | 0.0008 |
| PP_Pre_ and Fractional Fall of Haemoglobin | -0.04 | 0.61 | 0.38 | 0.018 |
| TPB_Pre_ and Haemoglobin nadir | 0.07 | 0.35 | -0.60 | 0.0001 |
| TPB_Pre_ and Fractional Fall of Haemoglobin | 0.01 | 0.93 | 0.44 | 0.005 |
| PMR and Haemoglobin nadir | 0.01 ^a^ | 0.94 | 0.05 ^b^ | 0.81 |
| PMR and Fractional Fall of Haemoglobin | -0.05 ^a^ | 0.53 | -0.27 ^b^ | 0.17 |
| PRR and Haemoglobin nadir | 0.05 ^c^ | 0.59 | 0.20 ^d^ | 0.35 |
| PRR and Fractional Fall of Haemoglobin | 0.01 ^c^ | 0.87 | -0.23 ^d^ | 0.26 |

n, number of participants; TPB_Pre_, pre-treatment total parasite burden; PP_Pre_, Pre-treatment peak parasitaemia; PMR, parasite multiplication rate; PRR, parasite reduction ratio.

a: n=155; b: n=27; c: n=142; d: n=25.

**Additional file 1: Table S10. Spearman’s correlations between parasite parameters and haemoglobin variables in participants inoculated with PfK13**

| Correlation | Pf K13 (n=15)* | |
| --- | --- | --- |
|  | **r-value** | **P-value** |
| PP_Pre_ and Haemoglobin nadir | -0.46 | 0.09 |
| PP_Pre_ and Fractional Fall of Haemoglobin | 0.66 | 0.007 |
| TPB_Pre_ and Haemoglobin nadir | -0.37 | 0.18 |
| TPB_Pre_ and Fractional Fall of Haemoglobin | 0.50 | 0.06 |
| PMR and Haemoglobin nadir | -0.49 | 0.04 |
| PMR and Fractional Fall of Haemoglobin | 0.61 | 0.016 |
| PRR and Haemoglobin nadir | 0.72 | 0.002 |
| PRR and Fractional Fall of Haemoglobin | -0.57 | 0.026 |

n, number of participants; TPB_Pre_, pre-treatment total parasite burden; PP_Pre_, Pre-treatment peak parasitaemia; PMR, parasite multiplication rate; PRR, parasite reduction ratio.

**References**

1. Marquart L, Baker M, O’Rourke P, McCarthy JS. Evaluating the pharmacodynamic effect of antimalarial drugs in clinical trials by quantitative PCR. Antimicrob Agents Chemother. **2015**; 59(7):4249–4259.

2. Moehrle JJ, Duparc S, Siethoff C, et al. First-in-man safety and pharmacokinetics of synthetic ozonide OZ439 demonstrates an improved exposure profile relative to other peroxide antimalarials. Br J Clin Pharmacol. **2013**; 75(2):535–548.

3. McCarthy JS, Griffin PM, Sekuloski S, et al. Experimentally induced blood-stage *Plasmodium vivax* infection in healthy volunteers. J Infect Dis. **2013**; 208(10):1688–1694.

4. McCarthy JS, Lotharius J, Rückle T, et al. Safety, tolerability, pharmacokinetics, and activity of the novel long-acting antimalarial DSM265: a two-part first-in-human phase 1a/1b randomised study. Lancet Infect Dis. **2017**; 17(6):626–635.

5. Stanisic DI, Gerrard J, Fink J, et al. Infectivity of *Plasmodium falciparum* in malaria-naive individuals is related to knob expression and cytoadherence of the parasite. Infect Immun.**2016**; 84(9):2689–2696.

6. Pasay CJ, Rockett R, Sekuloski S, et al. Piperaquine monotherapy of drug-susceptible *Plasmodium falciparum* infection results in rapid clearance of parasitemia but is followed by the appearance of gametocytemia. J Infect Dis.**2016**; 214(1):105–113.

7. Smith CM, Jerkovic A, Truong TT, Foote SJ, McCarthy JS, McMorran BJ. Griseofulvin impairs intraerythrocytic growth of *Plasmodium falciparum* through ferrochelatase inhibition but lacks activity in an experimental human infection study. Sci Rep. **2017**; 7:41975.

8. Griffin P, Pasay C, Elliott S, et al. Safety and reproducibility of a clinical trial system using induced blood stage Plasmodium vivax infection and its potential as a model to evaluate malaria transmission. PLoS Negl Trop Dis.**2016**; 10(12):e0005139.

9. McCarthy JS, Ruckle T, Djeriou E, Cantalloube C, Ter-Minassian D, Baker M. A Phase II pilot trial to evaluate safety and efficacy of ferroquine against early Plasmodium falciparum in an induced blood-stage malaria infection study. Malar J. **2016**; 15.

10. Krause A, Dingemanse J, Mathis A, Marquart L, Mohrle JJ, McCarthy JS. Pharmacokinetic/pharmacodynamic modelling of the antimalarial effect of Actelion-451840 in an induced blood stage malaria study in healthy subjects. Br J Clin Pharmacol; 2016;82.

11. Collins KA, Wang CYT, Adams M, et al. A *Plasmodium vivax* experimental human infection model for evaluating efficacy of interventions. J Clin Invest. **2020**; 130(6):2920–2927.

12. Phumla S, Cristina D, Hilary J, et al. Safety, tolerability, pharmacokinetics, and antimalarial activity of the novel Plasmodium phosphatidylinositol 4-Kinase inhibitor MMV390048 in healthy volunteers. Antimicrob Agents Chemother.**2021**; 64(4):e01896-19.

13. McCarthy JS, Rückle T, Elliott SL, et al. A single-dose combination study with the experimental antimalarials artefenomel and DSM265 to determine safety and antimalarial sctivity against blood-stage *Plasmodium falciparum* in healthy volunteers. Antimicrob Agents Chemother. **2019**; 64(1):e01371-19.

14. Collins KA, Wang CY, Adams M, et al. A controlled human malaria infection model enabling evaluation of transmission-blocking interventions. J Clin Invest. **2018**; 128(4):1551–1562.

15. McCarthy JS, Abd-Rahman AN, Collins KA, et al. Defining the antimalarial activity of Cipargamin in healthy volunteers experimentally infected with blood-stage *Plasmodium falciparum*. Antimicrob Agents Chemother. **2021**; 65(2):e01423-20.

16. Collins KA, Rückle T, Elliott S, et al. DSM265 at 400 milligrams clears asexual stage parasites but not mature gametocytes from the blood of healthy subjects experimentally infected with *Plasmodium falciparum*. Antimicrob Agents Chemother .**2019**; 63(4):e01837-18.

17. Collins KA, Abd-Rahman AN, Marquart L, et al. Antimalarial activity of artefenomel against asexual parasites and transmissible gametocytes during experimental blood-stage *Plasmodium vivax* Infection. J Infect Dis. **2020**; .doi.org/10.1093/infdis/jiaa287

18. Gaur AH, McCarthy JS, Panetta JC, et al. Safety, tolerability, pharmacokinetics, and antimalarial efficacy of a novel *Plasmodium falciparum* ATP4 inhibitor SJ733: a first-in-human and induced blood-stage malaria phase 1a/b trial. Lancet Infect Dis. 2020

19. McCarthy JS, Donini C, Chalon S, et al. A Phase 1, Placebo-controlled, randomized, single ascending dose study and a volunteer infection study to characterize the safety, pharmacokinetics, and antimalarial activity of the Plasmodium phosphatidylinositol 4-kinase inhibitor MMV390048. Clin Infect Dis. **2020**; 71(10):e657–e664.

20. Watts RE, Odedra A, Marquart L, et al. Safety and parasite clearance of artemisinin-resistant Plasmodium falciparum infection: A pilot and a randomised volunteer infection study in Australia. Seidlein L von, editor. PLOS Med. **2020**; 17(8):e1003203

21. First-in-human trial of single ascending dose, multiple ascending dose and malaria challenge model in healthy subjects. ClinicalTrials.gov. Cited 8 Jun 2021. https://clinicaltrials.gov/ct2/show/NCT03261401

22. Odedra A, Mudie K, Kennedy G, et al. Safety and feasibility of apheresis to harvest and concentrate parasites from subjects with induced blood stage Plasmodium vivax infection. Malar J. **2021**; 20(1):43.

23. ANZCTR Trial ID: NCT03542149. Phase 1b to assess safety, tolerability, pharmacokinetic profile, and antimalarial activity of single doses of co-administered OZ439 and PQP against early *Plasmodium falciparum* blood stage infection in healthy adult volunteers. 2018.

24. ANZCTR Trial ID: ACTRN12619001215112. Open label study using the *P. falciparum* induced blood stage malaria (IBSM) model to determine the safety and tolerablity and to characterise the antimalarial activity of a single-dose oral administration of ZY-19489
